# Supplementary material for: Modeling glioblastoma heterogeneity as a dynamic network of cell states
Source: Mol Syst Biol. 2021 Sep 16;17(9):e10105. doi: 10.15252/msb.202010105 (PMC8444284; doi:10.15252/msb.202010105)
Supplement: Supplementary file 5 — Source Data for Figure 3 [file MSB-17-e10105-s001.zip › Figure3A_sourcedata/GSEA_3065/hallmarks_state3.GseaPreranked.1623416274744/HALLMARK_PI3K_AKT_MTOR_SIGNALING.html]

Details for gene set HALLMARK\_PI3K\_AKT\_MTOR\_SIGNALING[GSEA]

|  || Dataset | state3 |
| Phenotype | NoPhenotypeAvailable |
| Upregulated in class | na\_neg |
| GeneSet | HALLMARK\_PI3K\_AKT\_MTOR\_SIGNALING |
| Enrichment Score (ES) | -0.45210257 |
| Normalized Enrichment Score (NES) | -1.5851581 |
| Nominal p-value | 0.0031007752 |
| FDR q-value | 0.01990304 |
| FWER p-Value | 0.178 |
Table: GSEA Results Summary

  

Fig 1: Enrichment plot: HALLMARK\_PI3K\_AKT\_MTOR\_SIGNALING      
 Profile of the Running ES Score & Positions of GeneSet Members on the Rank Ordered List

  

| PROBE | GENE SYMBOL | GENE\_TITLE | RANK IN GENE LIST | RANK METRIC SCORE | RUNNING ES | CORE ENRICHMENT || 1 | HSP90B1 |  |  | 7 | 0.563 | 0.0553 | No |
| 2 | CALR |  |  | 30 | 0.473 | 0.1000 | No |
| 3 | CLTC |  |  | 177 | 0.296 | 0.1127 | No |
| 4 | SLC2A1 |  |  | 587 | 0.188 | 0.0843 | No |
| 5 | CDK1 |  |  | 915 | 0.147 | 0.0613 | No |
| 6 | ITPR2 |  |  | 1114 | 0.129 | 0.0513 | No |
| 7 | ACACA |  |  | 1412 | 0.108 | 0.0277 | No |
| 8 | TSC2 |  |  | 1449 | 0.106 | 0.0341 | No |
| 9 | MAPK10 |  |  | 1652 | 0.094 | 0.0203 | No |
| 10 | ADCY2 |  |  | 1923 | 0.080 | -0.0029 | No |
| 11 | NCK1 |  |  | 1998 | 0.076 | -0.0039 | No |
| 12 | PRKAA2 |  |  | 2113 | 0.071 | -0.0099 | No |
| 13 | EGFR |  |  | 2416 | 0.060 | -0.0387 | No |
| 14 | VAV3 |  |  | 2539 | 0.056 | -0.0473 | No |
| 15 | CXCR4 |  |  | 2543 | 0.055 | -0.0421 | No |
| 16 | TNFRSF1A |  |  | 2695 | 0.050 | -0.0545 | No |
| 17 | STAT2 |  |  | 2749 | 0.049 | -0.0557 | No |
| 18 | PPP2R1B |  |  | 3116 | 0.038 | -0.0941 | No |
| 19 | PTEN |  |  | 3464 | 0.029 | -0.1313 | No |
| 20 | GSK3B |  |  | 3606 | 0.025 | -0.1450 | No |
| 21 | PIKFYVE |  |  | 3734 | 0.022 | -0.1575 | No |
| 22 | PIK3R3 |  |  | 3885 | 0.018 | -0.1730 | No |
| 23 | SMAD2 |  |  | 3913 | 0.017 | -0.1744 | No |
| 24 | E2F1 |  |  | 3983 | 0.016 | -0.1808 | No |
| 25 | PLCG1 |  |  | 4262 | 0.008 | -0.2120 | No |
| 26 | MAP2K3 |  |  | 4277 | 0.007 | -0.2129 | No |
| 27 | PLA2G12A |  |  | 4428 | 0.003 | -0.2299 | No |
| 28 | IRAK4 |  |  | 4944 | -0.009 | -0.2884 | No |
| 29 | TBK1 |  |  | 4992 | -0.011 | -0.2927 | No |
| 30 | PAK4 |  |  | 5002 | -0.011 | -0.2926 | No |
| 31 | MAP2K6 |  |  | 5029 | -0.012 | -0.2945 | No |
| 32 | RIPK1 |  |  | 5070 | -0.013 | -0.2978 | No |
| 33 | RPS6KA3 |  |  | 5318 | -0.019 | -0.3244 | No |
| 34 | NFKBIB |  |  | 5335 | -0.019 | -0.3243 | No |
| 35 | RAF1 |  |  | 5375 | -0.020 | -0.3268 | No |
| 36 | MAPK9 |  |  | 5418 | -0.021 | -0.3295 | No |
| 37 | RIT1 |  |  | 5453 | -0.023 | -0.3312 | No |
| 38 | CDKN1B |  |  | 5783 | -0.032 | -0.3659 | No |
| 39 | DUSP3 |  |  | 5793 | -0.033 | -0.3637 | No |
| 40 | RPS6KA1 |  |  | 5796 | -0.033 | -0.3606 | No |
| 41 | GRK2 |  |  | 5819 | -0.033 | -0.3599 | No |
| 42 | MAPK8 |  |  | 5826 | -0.034 | -0.3572 | No |
| 43 | EIF4E |  |  | 5835 | -0.034 | -0.3548 | No |
| 44 | TIAM1 |  |  | 5861 | -0.035 | -0.3542 | No |
| 45 | CDK2 |  |  | 5871 | -0.035 | -0.3517 | No |
| 46 | ATF1 |  |  | 5972 | -0.038 | -0.3594 | No |
| 47 | ACTR3 |  |  | 6122 | -0.044 | -0.3722 | No |
| 48 | PTPN11 |  |  | 6275 | -0.049 | -0.3849 | No |
| 49 | ACTR2 |  |  | 6282 | -0.049 | -0.3807 | No |
| 50 | CAB39 |  |  | 6565 | -0.059 | -0.4073 | No |
| 51 | PRKAG1 |  |  | 6621 | -0.062 | -0.4074 | No |
| 52 | MYD88 |  |  | 6658 | -0.064 | -0.4052 | No |
| 53 | MKNK2 |  |  | 6833 | -0.072 | -0.4181 | No |
| 54 | DDIT3 |  |  | 6918 | -0.077 | -0.4201 | No |
| 55 | MAPK1 |  |  | 6941 | -0.078 | -0.4149 | No |
| 56 | ECSIT |  |  | 7003 | -0.081 | -0.4138 | No |
| 57 | RALB |  |  | 7297 | -0.100 | -0.4376 | No |
| 58 | UBE2N |  |  | 7424 | -0.110 | -0.4411 | Yes |
| 59 | MAP3K7 |  |  | 7461 | -0.113 | -0.4340 | Yes |
| 60 | GRB2 |  |  | 7509 | -0.118 | -0.4276 | Yes |
| 61 | AKT1S1 |  |  | 7527 | -0.120 | -0.4176 | Yes |
| 62 | PRKAR2A |  |  | 7575 | -0.125 | -0.4106 | Yes |
| 63 | AKT1 |  |  | 7627 | -0.131 | -0.4035 | Yes |
| 64 | MAPKAP1 |  |  | 7733 | -0.144 | -0.4012 | Yes |
| 65 | PPP1CA |  |  | 8137 | -0.213 | -0.4264 | Yes |
| 66 | CDK4 |  |  | 8168 | -0.220 | -0.4079 | Yes |
| 67 | UBE2D3 |  |  | 8174 | -0.221 | -0.3865 | Yes |
| 68 | SQSTM1 |  |  | 8176 | -0.221 | -0.3646 | Yes |
| 69 | ARHGDIA |  |  | 8218 | -0.233 | -0.3460 | Yes |
| 70 | PIN1 |  |  | 8225 | -0.234 | -0.3234 | Yes |
| 71 | YWHAB |  |  | 8309 | -0.260 | -0.3070 | Yes |
| 72 | ARPC3 |  |  | 8311 | -0.260 | -0.2812 | Yes |
| 73 | AP2M1 |  |  | 8381 | -0.287 | -0.2605 | Yes |
| 74 | HRAS |  |  | 8396 | -0.293 | -0.2329 | Yes |
| 75 | ARF1 |  |  | 8459 | -0.321 | -0.2080 | Yes |
| 76 | CSNK2B |  |  | 8485 | -0.332 | -0.1778 | Yes |
| 77 | CDKN1A |  |  | 8657 | -0.461 | -0.1515 | Yes |
| 78 | RAC1 |  |  | 8673 | -0.484 | -0.1050 | Yes |
| 79 | CFL1 |  |  | 8698 | -0.516 | -0.0563 | Yes |
| 80 | PFN1 |  |  | 8732 | -0.624 | 0.0022 | Yes |
Table: GSEA details [plain text format]

  

Fig 2: HALLMARK\_PI3K\_AKT\_MTOR\_SIGNALING: Random ES distribution      
 Gene set null distribution of ES for **HALLMARK\_PI3K\_AKT\_MTOR\_SIGNALING**
